# DNA-loaded targeted nanoparticles as a safe platform to produce exogenous proteins in tumor B cells

**DOI:** 10.3389/fimmu.2024.1509322

**Published:** 2025-01-22

**Authors:** Maria Cristina Grimaldi, Sara Bozzer, Dick J. Sjöström, Linnea I. Andersson, Tom Eirik Mollnes, Per H. Nilsson, Luca De Maso, Federico Riccardi, Michele Dal Bo, Daniele Sblattero, Paolo Macor

**Affiliations:** ^1^ Department of Life Sciences, University of Trieste, Trieste, Italy; ^2^ Experimental and Clinical Pharmacology Unit, Centro di Riferimento Oncologico di Aviano (CRO), IRCCS, Aviano, Italy; ^3^ Linnaeus Centre for Biomaterials Chemistry, Linnaeus University, Kalmar, Sweden; ^4^ Department of Chemistry and Biomedical Sciences, Linnaeus University, Kalmar, Sweden; ^5^ Department of Immunology, Oslo University Hospital and University of Oslo, Oslo, Norway; ^6^ Research Laboratory, Nordland Hospital, Bodo, Norway

**Keywords:** polymeric nanoparticles, targeting, safety, *in vivo* transfection, zebrafish

## Abstract

**Introduction:**

The functionalization of nanoparticles (NPs) with an antiCD19 targeting mechanism represents a promising approach for the selective delivery of drugs and nucleic acids into normal and tumor B cells. This strategy has the advantage of minimizing off-target effects by restricting gene delivery to the desired cell population. However, the nanoplatform must guarantee both the local production of the protein and the safety of the treatment to allow an effective therapy with reduced systemic toxicity.

**Methods:**

In order to ensure a selective delivery of nucleic acids, we developed poly(lactic-co-glycolic acid) (PLGA)-poly(vinyl alcohol) (PVA) NPs loaded with an Enhanced Green Fluorescent Protein (EGFP)-coding plasmid and covalently coated with antiCD19 recombinant antibody as a targeting mechanism. To assess the functionality of the NPs, physicochemical characterization, safety tests, and transfection assay were employed to evaluate the NPs’ behavior *in vitro* and *in vivo*, in a human/zebrafish lymphoma xenograft model.

**Results:**

The results demonstrated that the PLGA-PVA nanoplatform was capable of efficiently encapsulating and releasing the payload. These nanostructures demonstrated a favorable safety profile, as evidenced by the absence of significant cell cytotoxicity, coagulation activation, complement system activation, and the slight activation of endothelial cells and leukocytes. The targeting mechanism facilitated the interaction of NPs with target cells, thereby enhancing their internalization and subsequent exogenous plasmid DNA (pDNA) translation and protein expression. In the human/zebrafish lymphoma xenograft model, no evidence of toxicity was observed, and targeted NPs demonstrated the capacity to enhance exogenous pDNA expression.

**Conclusion:**

Our findings provide a rationale for the use of targeted NPs as a DNA delivery system for the local expression of therapeutic proteins.

## 1 Introduction

Advances in cell and gene therapies have enabled the treatment of a wide range of conditions, from congenital disorders to solid cancers, leading to the approval of several gene therapy products in recent years. Gene therapy has become a clinical reality with the approval of advanced therapy medicinal products for treating distinct monogenetic diseases and B-cell malignancies (1). A concrete example includes oligonucleotide-based therapies such as Spinraza which is used to treat Spinal Muscular Atrophy (SMA). This therapy aims to increase intracellular protein production in motor neurons after intrathecal drug injection (2). Other examples include cell therapies such as Kymriah and Yescarta, for the treatment of acute lymphoblastic leukemia (ALL), and diffuse large B-cell lymphoma (DLBCL), respectively, producing a chimeric antigen receptor on T cell surface after an *ex vivo* viral transfection (3). *In vivo* gene therapies also include Luxturna, a surgical injection beneath the retina that provide a functioning RPE65 gene via adeno-associated viruses for the treatment of retinitis pigmentosa, and Zolgensma, which delivers a novel SMN gene to motor neuron cells via adeno-associated viruses following a local injection for SMA (4). RNA vaccines also offer the possibility of delivering genetic information for protein production, using liposomes or other nanostructures as transfection agents (5). Furthermore, our group has proposed a potential method for reducing the inflammatory process in an arthritic rat model via the local production of a recombinant antibody, neutralizing the complement system, obtained through the intraarticular injection of liposomes and a pDNA (6).

In all of these approaches, the specificity of the action is guaranteed by the localized injection of the delivery system, which avoids or reduces diffusion in the body, facilitates fast elimination, and prevents immune response and systemic side effects that are favored by an injection in the circulation (7). Furthermore, different strategies have been included to increase the efficacy of nucleic acid delivery *in vivo* and to avoid poor cellular uptake when injected in naked form. While viral methods ensure high gene transfection efficiency, their clinical applications are limited by the potential risk of inducing neoplastic transformation of transfected cells and a lack of specificity for target tissues, which precludes their general use via intravenous injection (8). Similarly, lipidic nanostructures are constrained by the induction of an immune response and a subsequent rapid elimination from the liver following intravenous injection (9–11). Moreover, their surface cannot be easily modified, to allow a specific delivery, linking targeting agents (12, 13).

In recent times, there has been a notable increase in interest surrounding non-viral vectors, particularly polymeric NPs, due to their enhanced potential as gene delivery platforms (14). They are non-immunogenic, can be easily modified by targeting ligands, and can be manufactured on a large scale (15). Biodegradable and biocompatible synthetic polymers are often used for NPs production due to their chemical versatility, high purity, and controlled manufacturing processes (16). Specifically, PLGA is a linear, amorphous aliphatic polyester approved by the Food and Drug Administration (FDA) and the European Medicines Agency (EMA) as a delivery system for therapeutic agents. *In vivo*, the payload release occurs because the polymer degrades by hydrolysis and reverts to its original monomers (i.e., lactic acid and glycolic acid), which are naturally formed under normal physiological conditions and then processed through metabolic pathways such as the Krebs cycle and excreted as CO_2_ and H_2_O. PLGA nanostructures are often stabilized with PVA as an emulsifier. PVA is a biodegradable, widely used water-soluble polymer that is non-toxic to living tissues (17, 18). These nanostructures can be easily targeted to specific tissues *in vitro* and *in vivo* after surface modification, such as the covalent binding of targeting mechanisms. In the context of B-cell malignancies, CD19 is a target that meets the criteria for specific recognition. It is overexpressed in nearly all tumoral B cells and shows rapid internalization upon binding a potential agent, such as antibodies (19, 20). When administered intravenously, NPs instantly encounter various components of the blood, including the cellular parts with erythrocytes, platelets, and leukocytes, as well as the humoral complement system, coagulation proteins, and other serum proteins. In particular, the activation of the complement system may cause the deposition of complement-activated products on NPs, causing their opsonization and consequent recognition by phagocytic cells (expressing complement receptors on their surface), including macrophages in liver, spleen, and other organs of the mononuclear phagocytic system (21, 22). This interaction has the potential to induce acute pathophysiological host responses, underscoring the importance of characterizing these interactions for the purpose of evaluating the potential health risks associated with novel NP-based therapeutics. Ideally, NPs should not induce hemolysis or adversely affect the function of erythrocytes. Furthermore, they should avoid recognition by any cell-bound and soluble immune receptors, in order to prevent a deleterious host immune response that may lead to thrombotic and inflammatory adverse effects, such as stroke (23).

In this study, we characterized both *in vitro* and *in vivo* the effectiveness and safety of antiCD19 targeted polymeric NPs loaded with a pDNA and capable of targeting CD19^+^ tumoral B cells with the objective of inducing the production of an exogenous protein.

## 2 Materials and methods

### 2.1 Production of PLGA-PVA NPs

PLGA-PVA NPs were synthesized by the double emulsion solvent evaporation method described by Vasir and Labhasetwar (24) with modifications. Briefly, PVA (Sigma-Aldrich Co., St Louis, MO, USA, molecular weight. 30,000-70,000, 87-90% hydrolyzed) was dissolved (final concentration 1 mg/mL) in MES buffered saline (0.1 M MES, 0.9% NaCl, pH 4.7). To covalently link the antiCD19 single chain Fragment variable-Fragment crystallizable (scFv-Fc, previously characterized in (25)) to PVA, 200 µL of the previous solution were activated with EDC and sulfo-NHS by incubation for 15 minutes at room temperature in a final volume of 1 mL. Subsequently, 300 µg of antiCD19 scFv-Fc (in a final volume of 1 mL) were added, and the mixture was incubated for 2 hours at room temperature to facilitate bond formation. The procedure was performed by exploiting the EDC/sulfo-NHS crosslinking protocol as manufacturer’s instructions (Thermo-Fisher Scientific, Waltham, MA, USA). The reaction was then purified using dialysis to remove unreacted components and then added to 4 mL of 2% (w/v) PVA solution in cold Tris-EDTA pH 8 (TE buffer). After, PLGA (30 mg, Sigma-Aldrich Co., molecular weight 30,000-60,000, lactide: glycolide 50:50) was dissolved in 1 mL of chloroform (Sigma-Aldrich Co.). To start the synthesis, 300 μL of TE buffer or FITC-Bovine Serum Albumin (FITC-BSA, 20 mg/mL, Sigma-Aldrich Co.) or pEGFP-N1 (1.7 mg/mL) were added to the PLGA solution and vortexed and sonicated (30 seconds at 15 W of the maximum power) with Bandelin Sonopuls HD 2070 (Bandelin electronic GmbH & Co. KG, BER, Germany) to form the primary emulsion. The obtained emulsion was added to the PVA solution and sonicated for 1 minute as described above to form a secondary emulsion. The resulting emulsion was stirred (750 rpm) overnight to allow for chloroform evaporation. NPs were collected by centrifugation at 12,000 g for 30 minutes at 4°C and the pellet was washed in TE buffer. Finally, NPs were resuspended in 2 mL of PBS filtered 0.2 μm and stored at 4°C. The production of untargeted NPs was conducted in accordance with the same protocol but skipping the antiCD19 scFv-Fc coupling step.

### 2.2 Dynamic light scattering

Particle size (average diameter), polydispersity index, and surface charge (zeta potential) were determined by the DLS technique using Zetasizer Nano ZSP (Malvern Panalytical, Malvern, UK). NPs were diluted 1:200 (v/v) in H_2_O MilliQ filtered 0.2 μm and measurements were done at 37°C and a scattering angle of 173°.

### 2.3 Scanning electron microscopy

NPs were diluted 1:50 (v/v) in H_2_O MilliQ filtered 0.2 μm. A drop of the sample was deposited on a glass coverslip and left to dry at room temperature. Glass coverslips were mounted on aluminum stubs coated with double-sided carbon tape. Samples were carbon coated using the Q150T ES plus sputter coater (Quorum Technologies, Laughton, UK). Samples were analyzed using a Gemini 300 SEM (Zeiss, Oberkochen, Germany, UE), working in secondary electron mode, at an acceleration voltage and a working distance of 2 kV and 3 mm.

### 2.4 Cryogenic electron microscopy

TEM grids (C-Flat 1.2/1.3 Cu 300, Protochips) were treated by glow discharge with PELCO easiGlow (Ted Pella, Inc., CA, USA) for 45 seconds and with 20 mA to make grids hydrophilic and mounted inside the Vitrobot instrument (Thermo-Fisher Scientific). NPs were diluted 1:100 (v/v) in PBS and 4 µL were deposited onto the grid. The Vitrobot automatically performed the plunge freezing by blotting: after a few microliters of NPs were adsorbed onto a grid, the sample was blotted with filter paper to make a thin aqueous layer, and then plunge frozen into liquid ethane for instant vitrification. The blotting was done at 20°C, with humidity of 95%, and for 3 seconds blot time. Then, TEM grids were placed inside the sample holder and kept at the temperature of liquid nitrogen. Finally, the grids were clamped and inserted in the Cryo-EM microscope Glacios (Thermo-Fisher Scientific) for the imaging.

### 2.5 Nanoparticle tracking analysis

NTA was performed using NanoSight LM10 (Malvern Panalytical, Lissone, Italy) diluting NPs 1:4000 (v/v) in H_2_O milliQ immediately before the measurements.

### 2.6 Evaluation of the antiCD19 scFv-Fc coating on the surface of NPs

ELISA wells were coated overnight at 4°C with a goat anti-human IgG antibody (Sigma-Aldrich Co., final concentration 2 ng/μL in a final volume of 100 μL/well). The unbound sites were blocked by static incubation with 2% skim milk in PBS for 1 hour at room temperature with 150 μL/well. Following three washes with PBS-Tween 20 0.1% and three with PBS, the plate was statically incubated for 1 hour at room temperature with different amounts (5-10-25 μL) of NPs in a final volume of 100 μL/well. Following washes, the presence of antiCD19 scFv-Fc was revealed through a goat anti-human IgG alkaline phosphatase-conjugated antibody (Sigma-Aldrich Co., final concentration 4 ng/μL) that was statically incubated for 1 hour at room temperature in 100 μL/well. After washes, the alkaline phosphatase substrate p-Nitrophenyl phosphate (pNPP, 1 mg/mL, Sigma-Aldrich Co.) in glycine buffer (0.1 M glycine, 0.1 mM MgCl_2_, and 0.1 mM ZnCl_2_, pH 9.6) was added to develop the reaction at 37°C. The optical density (OD) was read at 405 nm with ELISA Reader TECAN Infinite M200 (Tecan Italia S.r.l., Milan, Italy).

### 2.7 Evaluation of the integrity and quantification of antiCD19 scFv-Fc coating on the surface of NPs

30 μL of NPs were centrifuged at 8,000 g for 5 minutes. Following the removal of the supernatant, NPs were resuspended in 15 μL of non-reducing 4x Laemmli Sample Buffer (Bio-Rad Laboratories, CA, USA) and boiled for 30 minutes. The positive control of purified antiCD19 scFv-Fc (300 ng) was diluted in the same buffer and then boiled for 30 minutes. Non-reducing conditions were chosen as the objective was to monitor the integrity and evaluate the correctness of the antiCD19 scFv-Fc assembly. Samples were resolved on SDS-polyacrylamide gel and transferred onto a nitrocellulose membrane (GE HealthCare, Chicago, IL, USA). Following a blocking step of 45 minutes with 2% skim milk in PBS at room temperature, the membrane was incubated for 1 hour at room temperature with a goat anti-human IgG alkaline phosphatase-conjugated antibody (Sigma-Aldrich Co., final concentration 2 ng/μL). After three washes, with PBS-Tween 20 0.1% and three with PBS, the membrane was developed with BCIP and NBT (Sigma-Aldrich Co.) in alkaline phosphatase buffer (100 mM Tris-HCl, 0.1 M NaCl, 5 mM MgCl_2_, pH 9.6). The same protocol was employed to evaluate the antiCD19 scFv-Fc coupling efficiency, with samples resuspended in reducing 4x Laemmli Sample Buffer. Reducing conditions were preferred to construct an antiCD19 scFv-Fc calibration curve. This approach was taken to ensure precision and reliability by ensuring that all proteins migrated according to their molecular mass without alteration due to the presence of aggregates. This is a critical step in obtaining a linear relationship between the amount of protein and the intensity of the band on the gel. The intensity of the bands was quantified using a ChemiDoc Imaging System (Bio-Rad Laboratories). The calibration curve was constructed using note amounts of purified antiCD19 scFv-Fc with the resulting band intensities plotted against the note amounts. The linear regression coefficient (R^2^) was determined to be 0.9994. The total amount of bound antiCD19 scFv-Fc was calculated by interpolation of the bands’ intensity relative to the samples of NPs to the calibration curve. The total number of antiCD19 scFv-Fc in the preparation was calculated using the molecular weight of the molecule. The number of bound antiCD19 scFv-Fc per NP was calculated by dividing the total number of antiCD19 scFv-Fc by the total number of NPs in the preparation, as determined by NTA.

### 2.8 Cell cultures

BJAB cells (Burkitt lymphoma cell line) and JURKAT cells (acute T cell leukemia cell line) were maintained in suspension in Roswell Park Memorial Institute 1640 (RPMI-1640, Sigma-Aldrich Co.) medium supplemented with 10% (v/v) fetal bovine serum (FBS, Sigma-Aldrich Co.), 100 μg/mL streptomycin (Sigma-Aldrich Co.), and 100 U/mL penicillin (Sigma-Aldrich Co.). Cells were incubated at 37°C in a humidified atmosphere with 5% CO_2_. Fresh medium was substituted every 48 hours.

### 2.9 Cell viability assay

BJAB or JURKAT (1000 cells/μL) were incubated for 24 hours at 37°C under shaking (800 rpm) with 10 μL of NPs in a final volume of 200 μL of RPMI-1640 supplemented medium. 20 µL of 3-(4,5-dimethylthiazol-2-yl)-2,5-diphenyltetrazolium bromide (MTT, 5 mg/mL, Sigma-Aldrich Co.) were subsequently added, and samples were incubated for 4 hours at 37°C under shaking conditions (800 rpm). Afterward, samples were centrifuged for 3 minutes at 20,000 g to precipitate all the formazan crystals. The supernatant was removed, and the deposited crystals were solubilized in 200 µL of dimethyl sulfoxide (DMSO, Sigma-Aldrich Co.). The OD was measured at a wavelength of 570 nm ELISA Reader TECAN Infinite M200 (Tecan Italia S.r.l.). The viability percentage was calculated using untreated cells as a reference point, with 100% represented by the untreated control group. This was expressed as percent of mitochondrial activity. The assay was performed in triplicate with each sample analyzed in triplicate. Controls are represented by untreated cells and cells treated with PBS (representing the buffer in which the NPs are resuspended).

### 2.10 CH50 screening assay

Functional activity of the complement system was performed by measuring complement hemolytic activity 50% (CH50) on sensitized mutton erythrocytes (EA, Microbiol S.r.l., Cagliari, CA, Italy) as previously described (21). Briefly, normal human serum (NHS, 100 μL) and NPs (8 μL) were incubated for 2 hours at 37°C under shaking conditions (750 rpm). Then samples were centrifuged for 5 minutes at 8,000 g, and the supernatants were diluted in Complement Fixation Diluent (CFD, 142 mM NaCl, 5 mM Na-5-5-diethylarbiturate, 0.5 mM MgCl_2_, 0.05% agar, 0.01% NaN_3_, 10 mM EGTA). The samples were then diluted 1:50, 1:100, 1:200, and 1:400 (v/v) to a final volume of 200 μL. Subsequently, 50 μL of EA 1% were added to each sample and incubated for 30 minutes to activate the classical complement pathway. The total lysis is represented by EA 1% diluted in H_2_O, while the blank is represented by EA 1% diluted in CFD. The reaction was immediately terminated by the addition of cold EDTA (final concentration 20 mM). The percent lysis was quantified at 415 nm with an ELISA Reader TECAN Infinite M200 (Tecan Italia S.r.l.) after centrifugation at 12,000 g for 1 minute. Complement activation was checked by preparing a standard curve using NHS as a positive control. The percentage of lysis was calculated using the following formula:


$$Lysis\;\left(\%\right)=\frac{OD\;415\;Sample-OD\;415\;Blank}{OD\;415\;Total\;lysis-OD\;415\;Blank}\times 100$$

The percentage of lysis was plotted against the serum dilution, and the dilution required for 50% hemolysis was calculated. The CH50 (U/mL) (Hemolytic Complement 50) provides the number of hemolytic units present in 1 mL of NHS.

### 2.11 Hemolytic assay

Direct erythrocyte cell lysis was performed by incubating 1% EA (Microbiol S.r.l.) with NPs (8 μL) diluted in CFD for 2 hours at 37°C under shaking conditions (750 rpm). The percentage of lysis was calculated as described in the paragraph “2.10 CH50 screening assay”.

### 2.12 Clotting test

NPs (4 μL/well), and normal human-citrate anticoagulated plasma (NHP, 80 μL/well) were seeded in a 96-well plate. To assess the clotting capacity of NPs, CaCl_2_ (final concentration 20 mM) was added to initiate the clotting reaction. The eventual coagulation resulted in an increase in the turbidity of the well, which was read at 405 nm every 2 minutes for 70 minutes with an ELISA Reader TECAN Infinite M200 (Tecan Italia S.r.l.). The half-coagulation time is defined as the point at which 50% of the coagulation process has occurred. The assay was performed in triplicate with each sample analyzed in triplicate.

### 2.13 Encapsulation efficiency and fluorescence quantification of NPs

The fluorescence signal corresponding to the FITC-BSA (maximum excitation/emission 495/519 nm) was acquired with ChemiDoc Imaging System (Bio-Rad Laboratories) and expressed as corrected total cell fluorescence (CTCF) calculated according to the following formula:


$$\begin{array}{c} CTCF=\text{IntegratedDensity}-[\left(\text{Area Region Of Interest ROI}\right)\\ \times (\text{Mean Fluorescence Intensity of Background})] \end{array}$$

The signal corresponding to the unencapsulated compound was interpolated with a FITC-BSA calibration curve to extrapolate the amount of FITC-BSA encapsulated. The encapsulation efficiency (EE%) was calculated indirectly using the following formula:


$$EE\;\left(\%\right)=\frac{Total\;amount\;of\;compound-unencapsulated\;compound}{Total\;amount\;of\;compound}\times 100$$

The fluorescence signal corresponding to the FITC-BSA loaded NPs was acquired with a ChemiDoc Imaging System (Bio-Rad Laboratories) immediately after synthesis and after 1 year. The data were expressed CTCF.

### 2.14 *In vitro* release profiles

The release profile of the FITC-BSA loaded NPs was analyzed in 50 μL of PBS or NHS diluted 1:10 in PBS, or cytosol mimic buffer (142 mM KCl, 5 mM NaCl, 5 mM MgCl_2_, 25 mM Hepes-KOH, pH 7.2, 1 mg/mL BSA, buffer composition from reference (26)) at 4°C or 37°C under shaking conditions (750 rpm) over 0.5-1 hours. Subsequently, the samples were centrifuged at 8,000 g for 5 minutes. Following this, a standard volume of the supernatant was collected, and a fixed volume was maintained by the addition of the same volume at each subsequent time point. The release was indirectly quantified by interpolating the fluorescent signal corresponding to the released compound with a FITC-BSA calibration curve thereby extrapolating the amount of FITC-BSA released as described in the paragraph “2.13 Encapsulation efficiency and fluorescence quantification of NPs”.

### 2.15 FITC-BSA fluorescence sensibility at different pH

FITC-BSA loaded NPs or FITC-BSA were diluted 1:10 (v/v) in 0.9% NaCl, pH 4-8 for 1 hour. The fluorescence signal was acquired using a ChemiDoc Imaging System (Bio-Rad Laboratories). The fluorescence variation was expressed as CTCF and quantified by setting the fluorescent signal at pH 8 (standard conditions) as the 100% reference.

### 2.16 Human whole blood and endothelial cell model for toxicity tests

Whole blood sampling, plasma preparation, human lung microvascular endothelial cells (HLMVECs; Cell Applications Inc, San Diego, CA) culturing, incubation of NPs on HLMVECs in combination with the human whole blood model, immunoassays, and flow cytometry tests were performed as described in (27). Whole blood sampling and analysis were performed according to the ethical guidelines from the Declaration of Helsinki. Informed written consent was obtained from all blood donors. The study was approved by the ethical committee of the Norwegian Regional Health Authority, ethical permit REK#S-04114, 2010/934. In brief, HLMVECs were cultured up to passage 5, before seeding to 48-well cell culture plates. After 2-3 days of daily growth medium exchanges, cells were washed with 37°C PBS and incubated with 300 μL of lepirudin-anticoagulated (final concentration 50 μg/mL) whole blood pre-mixed with 60 μL NPs for 4 hours at 37°C, 5% CO_2_ and 50 rpm shaking. 10 μL whole blood was isolated after 15 minutes for monocyte and granulocyte flow cytometry. EDTA was added to a final concentration of 20 μM after sampling. After 4 hours of incubation, whole blood was isolated and supplemented with EDTA (final concentration 20 mM). One 10 μL-aliquot whole blood was isolated for platelet flow cytometry, also supplemented with 10x CTAD (8 mM trisodium citrate, 1.1 M theophylline, 26 mM adenosine, 14 mM dipyridamole; Greiner Bio-One, Kremsmünster, Austria) to limit any further platelet activation. Plasma was prepared from the remaining whole blood by centrifugation at 3,000 g for 15 minutes at 4°C. All plasma samples were kept at -80°C before immunoassays. Granulocytes, monocytes, platelets, and HLMVECs were analyzed on an Accuri C6 (BD Biosciences, Franklin Lakes, NJ) and data was analyzed with FlowJo, version 10 (Tree Star, Ashland, OR). Immunoassays on plasma were conducted to detect the complement activation markers C3b/C3c, as a marker of C3-cleavage, and the soluble terminal complement complex (sC5b-9). This was achieved through the use of specific antibodies directed against a neoepitope of human C3, which was expressed on the cleavage fragments C3b/C3c/iC3b and the terminal complement complex, respectively, previously described in (28–30). Platelet Factor 4 (PF-4) and Neutrophil-Activating Peptide 2 (NAP-2) were analyzed with ELISA DuoSets (R&D Systems, Minneapolis, MN). Tecan plate reader Sunrise (Tecan Nordic, Stockholm, Sweden) was used for absorbance measurement, and Magellan software, version 7.1 (Tecan Nordic AB), was used for data analysis. Cytokines IL-1β, IL-6, IL-8, and Tumor Necrosis Factor (TNF) with a 4-plex kit (R&D systems). Blood cell counts were acquired with Swelab Alfa blood cell counter (Boule International, Spånga, Sweden) according to the manufacturer’s instructions.

### 2.17 Binding and internalization studies of NPs

BJAB or JURKAT (250,000 cells) were resuspended in 1 mL of RPMI-1640 supplemented medium and incubated at 37°C for 1 hour in rotation with 10 μL of FITC-BSA loaded NPs. For the internalization studies, cells were treated with 100 μL of Pronase (2 mg/mL, Sigma-Aldrich Co.) for 30 minutes at 4°C. The interaction between the cells and NPs was evaluated by flow cytometric analysis performed with an Attune^®^ NxT Acoustic Focusing flow cytometer (Thermo Fisher Scientific), acquiring 10,000 events; data were analyzed with Attune NxT Software. The percentage of positive cells with only bound NPs was calculated by subtracting the percentage of positive cells with only internalized NPs from that of positive cells with bound and internalized NPs. Similarly, the Mean Fluorescence Intensity (MFI) of only internalized NPs was subtracted from that of bound and internalized NPs thereby obtaining the MFI of only the bound NPs.

### 2.18 *In vitro* transfection studies

BJAB or JURKAT (250,000 cells) were resuspended in 400 μL of RPMI-1640 supplemented medium and treated with NPs at a volume corresponding to 3 μg of encapsulated pDNA, at 37°C and 5% CO_2_. Following a 24-hour incubation period, cells were washed with PBS and incubated for a further 96 hours with fresh medium. Transfection efficiency was evaluated by flow cytometric analysis performed on an Attune NxT Acoustic Focusing flow cytometer (Thermo Fisher Scientific), acquiring 10,000 events; data were analyzed with Attune NxT Software.

### 2.19 Zebrafish embryos handling and maintenance

Fertilized zebrafish eggs were placed in E3 Medium (5 mM NaCl, 0.17 mM KCl, 0.33 mM CaCl_2_, 0.33 mM MgSO_4_) supplemented with 0.5% methylene blue and incubated at 28°C. At 24 hours post-fertilization (hpf), the eggs were manually dechorionated and placed in E3 Medium supplemented with Phenylthiourea (PTU, final concentration 0.2 mM) to prevent melanogenesis and thus the pigmentation of the embryos. Zebrafish embryos were handled according to standard rules and procedures for animal wellness “https://zfin.org accessed on 01 June 2022”. All experimental procedures involving animals were executed after Ministerial Approval NO1832SBL22.

### 2.20 Human/zebrafish lymphoma xenograft model

Zebrafish embryos were anesthetized using tricaine (Sigma-Aldrich Co., final concentration 0.02%) at 48 hpf and placed on agarose plates, after which the excess water was removed to facilitate injection. BJAB cells were labeled with Calcein Red Orange-AM (Calcein-AM, Thermo Fisher Scientific, maximum excitation/emission 577/590 nm) for the biodistribution studies according to the manufacturer’s instructions. A total of approximately 2,500 labeled BJAB cells were injected into each embryo, with a final volume of 9.2 nL in the perivitelline space using capillary glasses and a Nanoject II Auto-Nanoliter Injector (Drummond Scientific Co., Broomall, PA, USA). The entire process was conducted using a SteREO Microscope Discovery.V8 (Zeiss). Following the injection of cells, embryos were maintained at 30°C and examined using the fluorescence microscope Nikon Eclipse Ti-E live system to ascertain the distribution of the injected cells. The images were subsequently analyzed using the ImageJ software.

### 2.21 Biodistribution studies of NPs

Biodistribution studies of NPs were performed by injecting FITC-BSA loaded NPs (4.6 nL/embryo) into the duct of Cuvier of human/zebrafish lymphoma xenograft models using capillary glasses and a Nanoject II Auto-Nanoliter Injector (Drummond Scientific Co.). At 24 hours post-injection (hpi), the biodistribution of the NPs was evaluated using a fluorescence microscope Nikon Eclipse Ti-E live system, and the resulting images were analyzed with ImageJ software. Subsequently, the fluorescence within the designated ROI was quantified for each embryo, and the CTCF was calculated as described in the paragraph “2.13 NPs encapsulation efficiency and fluorescence quantification”.

### 2.22 Agarose gel electrophoresis

pDNA topology and encapsulation by NPs were investigated by agarose gel electrophoresis. NPs were resuspended 1:1 (v/v) in chloroform (Sigma-Aldrich Co.) and incubated for 90 minutes at 37°C under shaking conditions (750 rpm). Samples were diluted 1:2 (v/v) with cold TE buffer, vortexed for 1 minute, and centrifuged for 5 minutes at 12,000 g. The aqueous phase which contained the pDNA, was recovered and subjected to electrophoresis on a 1% agarose gel in TAE buffer (40 mM Tris, 1 mM EDTA, 20 mM acetate, pH 8.6) containing SYBR Safe DNA gel staining dye (Thermo Fisher Scientific), for 30 minutes. pDNA was visualized with a ChemiDoc Imaging System (Bio-Rad Laboratories).

### 2.23 Gene expression studies of EGFP after *in vitro* transfection studies

Following a 24-hour incubation period with a volume of NPs corresponding to 3 μg of encapsulated pDNA, total RNA was extracted from BJAB and JURKAT cells with TRIzol reagent (Life Technologies, Carlsbad, CA, USA). Subsequently, 1 μg of RNA was reverse transcribed using Xpert cDNA Synthesis Supermix (GRiSP, Porto, Portugal) according to the manufacturer’s instructions. The expression of the EGFP gene was conducted through quantitative RT-PCR (qRT-PCR) using PowerTrack SYBR Green Master Mix in a CFX96 Real-Time PCR System (Bio-Rad Laboratories). Quantitative Real-Time conditions were as follows: 95°C for 3 minutes; 40 cycles at 95°C for 10 seconds and at 60°C for 30 seconds. The human L34 (*RPL34*) transcript was selected as the normalizer, and the relative abundance of the EGFP transcript was determined using the comparative ΔΔCt method. Primer pairs were designed using the open-source Primer3 program version 4.1.0 (https://primer3.ut.ee/), to generate an amplicon of 135 (*EGFP*), and 166 (*RPL34)* bp, and to have a melting temperature (tm) of approximately 60°C. The sequences of the primers are provided in **Supplementary Table 2**.

### 2.24 Gene expression studies of EGFP after *in vivo* transfection

Zebrafish embryos, previously injected with approximately 2,500 BJAB cells, were administered with a volume of NPs (corresponding to 0.8 ng of encapsulated pDNA) in the duct of Cuvier. At 24 hpi, zebrafish embryos were euthanized with 0.02% tricaine. Total RNA was extracted from experimental groups of 30 embryos with TRIzol^®^ reagent (Life Technologies). Subsequently, 1 μg of RNA was reverse transcribed using Xpert cDNA Synthesis Supermix (GRiSP) according to the manufacturer’s instructions. The expression of the EGFP gene was conducted through qRT-PCR using PowerTrack SYBR Green Master Mix in a CFX96 Real-Time PCR System (Bio-Rad Laboratories). Quantitative Real-Time conditions were as follows: 95°C for 3 minutes; 40 cycles at 95°C for 10 seconds and at 60°C for 30 seconds. Zebrafish beta-actin (*β-actin*) was selected as the normalizer, and the relative abundance of the EGFP transcript was determined using the comparative ΔΔCt method. The data are expressed as a fold change ratio between samples treated solely with NPs and samples treated with NPs and BJAB cells. Primer pairs were designed using the open-source Primer3 program version 4.1.0 (https://primer3.ut.ee/) to generate an amplicon of 135 (*EGFP*), and 300 (*β-actin*) bp, and to have a melting temperature (tm) of about 60°C. Primers’ sequences are listed in **Supplementary Table 2**.

### 2.25 Statistical analysis

Measures of the mean, standard deviation (SD), standard error of the mean (SEM), biological replicates, and statistical analyses are indicated in each figure legend. The data were analyzed using GraphPad (San Diego, CA) Prism version 9 by one-way ANOVA with Dunnett multiple-comparison test for the comparison of multiple columns and t-tests for the comparison of two columns. A p-value < 0.05 was considered statistically significant and shown in the figures.

## 3 Results

### 3.1 Synthesis and characterization of untargeted and antiCD19 scFv-Fc targeted nanoplatforms

**Figure 1:**
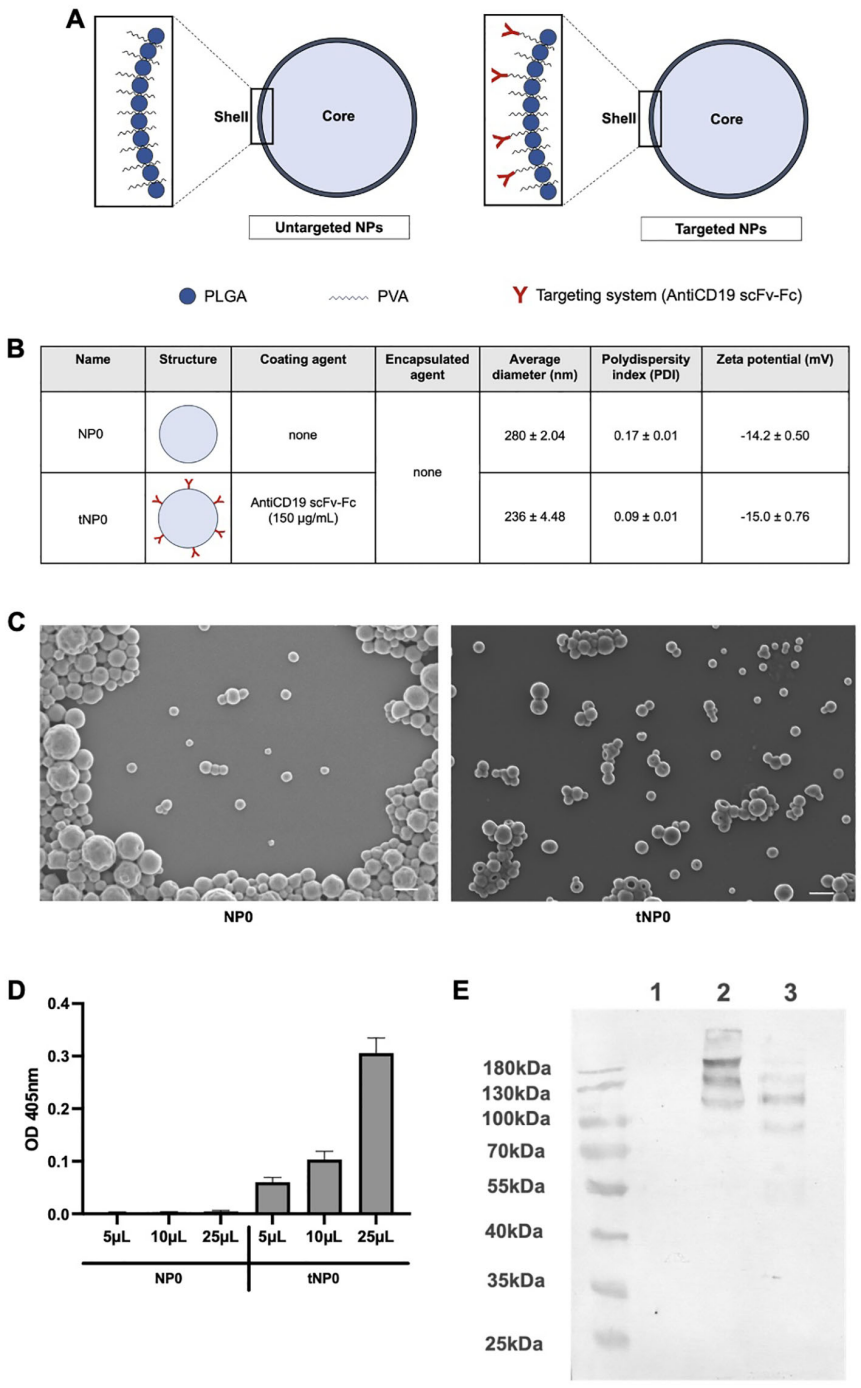
Structure and characterization of NPs. **(A)** Schematic representation of untargeted NP (NP0, left) and antiCD19 targeted NP (tNP0, right). NP0 are composed by an aqueous inner core (light blue) and a polymeric shell in PLGA (blue sphere) and PVA (black wavy line). In tNP0 the polymeric shell is composed of PLGA and PVA which is covalently linked to the antiCD19 targeting mechanism (red). **(B)** Summary of the composition and features of NP0 and tNP0 obtained by DLS. **(C)** SEM images of NP0 and tNP0 (scale bar 300 nm). **(D)** ELISA test performed on different amounts of NP0 (as negative control) and tNP0 incubated with a goat anti-human IgG alkaline phosphatase-conjugated to detect the presence of antiCD19. The values are shown as mean ± SD of n=2. **(E)** Western blot conducted in non-reducing condition to evaluate the integrity of the antiCD19. NP0 (Lane 1), tNP0 (Lane 2) and 300 ng of purified antiCD19 (Lane 3) show the expected molecular weight (110 kDa).

Untargeted (NP0) and targeted (tNP0) NPs were synthesized via a standardized double emulsion solvent evaporation method with an NTA yield of approximately 2x10^12^ NPs/mL. The structures are illustrated in **Figure 1A**. Both preparations consisted of an aqueous inner core (comprising of TE buffer for these formulations) and an outer shell in PLGA and PVA. In tNP0, the shell was covalently covered by a recombinant antiCD19 scFv-Fc as a targeting mechanism (defined as antiCD19 (25)). These particles were analyzed *in vitro* and the resulting data regarding their physicochemical parameters, including size, polydispersity index (PDI), and charge, are summarized in **Figure 1B**. NPs exhibited an average diameter of less than 300 nm (280 ± 2.04 nm for NP0 and 236 ± 4.48 nm for tNP0). Additionally, NPs exhibited a homogeneous distribution, as indicated by the low PDI values (0.17 ± 0.01 for NP0 and 0.09 ± 0.01 for tNP0). Both NP0 and tNP0 have a negative surface charge (-14 ± 0.50 mV and -15 ± 0.76 mV, as zeta potential respectively). The spherical shape of the NPs was verified by SEM imaging (**Figure 1C**). The results of the size were consistent with those obtained by DLS. Furthermore, SEM images indicated that the conjugation of antiCD19 on the surface of NPs did not affect the morphological characteristics, preserving the spherical shape of NP0, which was also observed for tNP0. Comparable results were obtained with NPs stored at 4°C for 1 year, demonstrating the stability of the nanoplatforms. The presence of the targeting mechanism in tNP0 was confirmed by ELISA and western blot. ELISA (**Figure 1D**) demonstrated the presence of the antiCD19 on the external shell, and the absence of signal from NP0 confirmed the specificity of the test. Instead, western blot (**Figure 1E**) allowed for the evaluation of antiCD19 integrity. The synthesis and conjugation process did not affect the antiCD19 structure, and bands were only visualized relative to the targeting mechanism covalently linked to polymers in tNP0, not in NP0. The antiCD19 exhibited the expected molecular weight of 110 kDa, as corroborated by the bands corresponding to this molecular weight in lanes 2 and 3. The bands representing higher molecular weights in lanes 2 and 3, respectively, were attributed to the molecule linked to the polymers and aggregates resulting from the sample preparation process. Furthermore, western blot was employed to quantify the amount of antiCD19 that was covalently linked to the surface of tNP0. The estimated binding density of antiCD19 on the surface of NPs was approximately 40 molecules per tNP0.

### 3.2 *In vitro* safety examination

**Figure 2:**
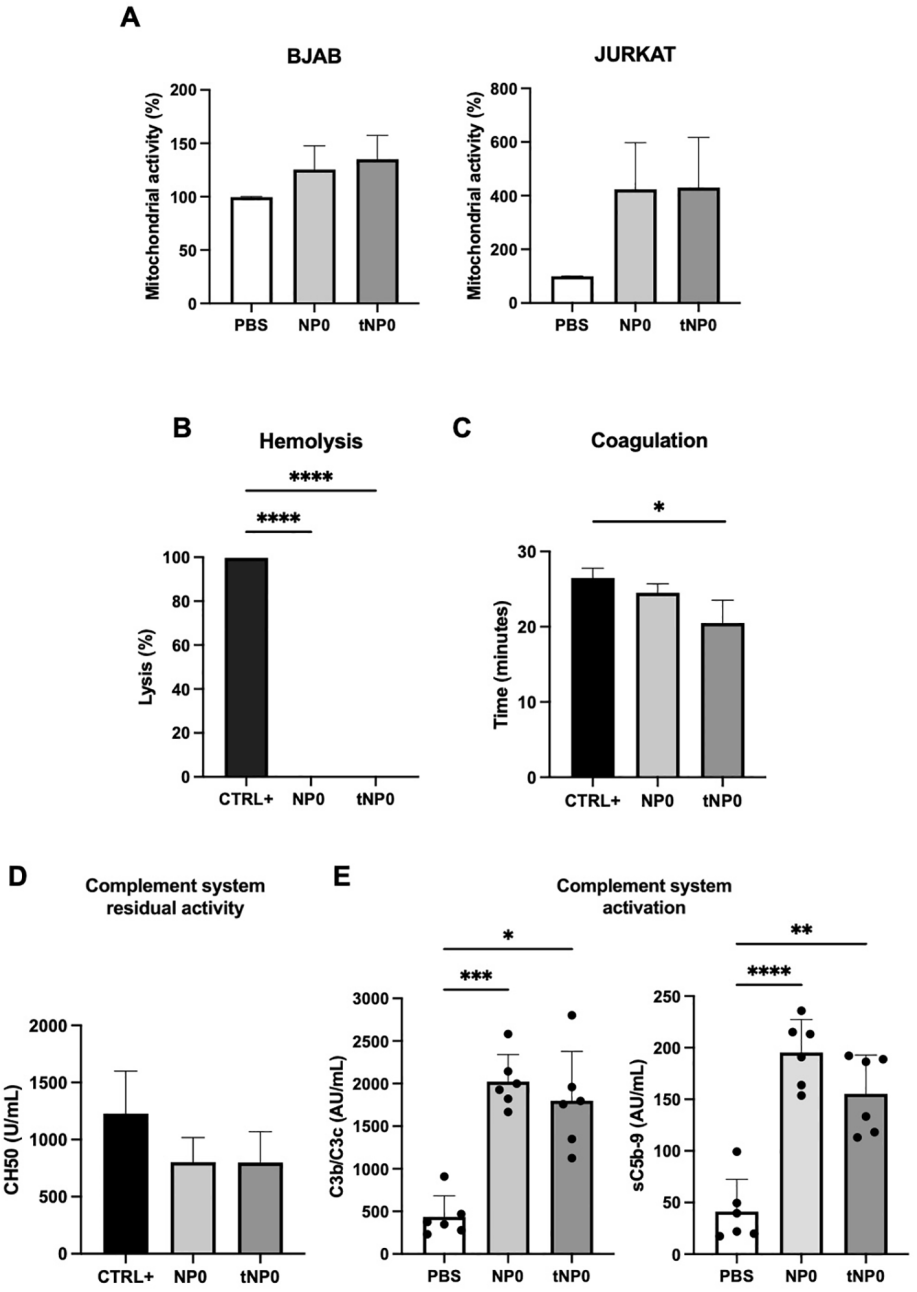
*In vitro* test for safety examination of NPs. **(A)** MTT cytotoxicity analysis of BJAB (left) and JURKAT (right) cell lines incubated with NP0 (10 μL) and tNP0 (10 μL) for 24 hours. Data are presented as % of mitochondrial activity compared to cells treated with PBS and reported as mean ± SD of n=3. **(B)** *In vitro* analysis of the direct hemolysis by 8 μL of NPs. Data are presented as % of lysis and compared to erythrocytes treated with H2O (CTRL+ as positive control) and reported as mean ± SD of n=3. Statistical was calculated by One-way ANOVA multiple comparison test. **** = P ≤ 0.0001. **(C)** Half-coagulation time expressed as NPs (4 μL) treated-plasma turbidity compared to a no treated one (CTRL+ as positive control), reported as mean ± SD of n=3. Statistical was calculated by One-way ANOVA multiple comparison test. * = P ≤ 0.05. **(D)** Complement system residual activity expressed as number of hemolytic units per mL (CH50) of NPs (8μL)-treated serum compared to a no treated one (CTRL+ as positive control), reported as mean ± SD of n=2. **(E)** Complement activation by NP0 (60 μL) and tNP0 (60 μL) in human whole blood evaluated by measuring the formation of C3b/C3c (left) and sC5b-9 (right) by ELISA. Sample treated with PBS as negative control. The values are shown as mean ± SD of n=6. Statistical was calculated by One-way ANOVA multiple comparison test. * = P ≤ 0.05; ** = P ≤ 0.01; *** = P ≤ 0.001; **** = P ≤ 0.0001.

**Figure 3:**
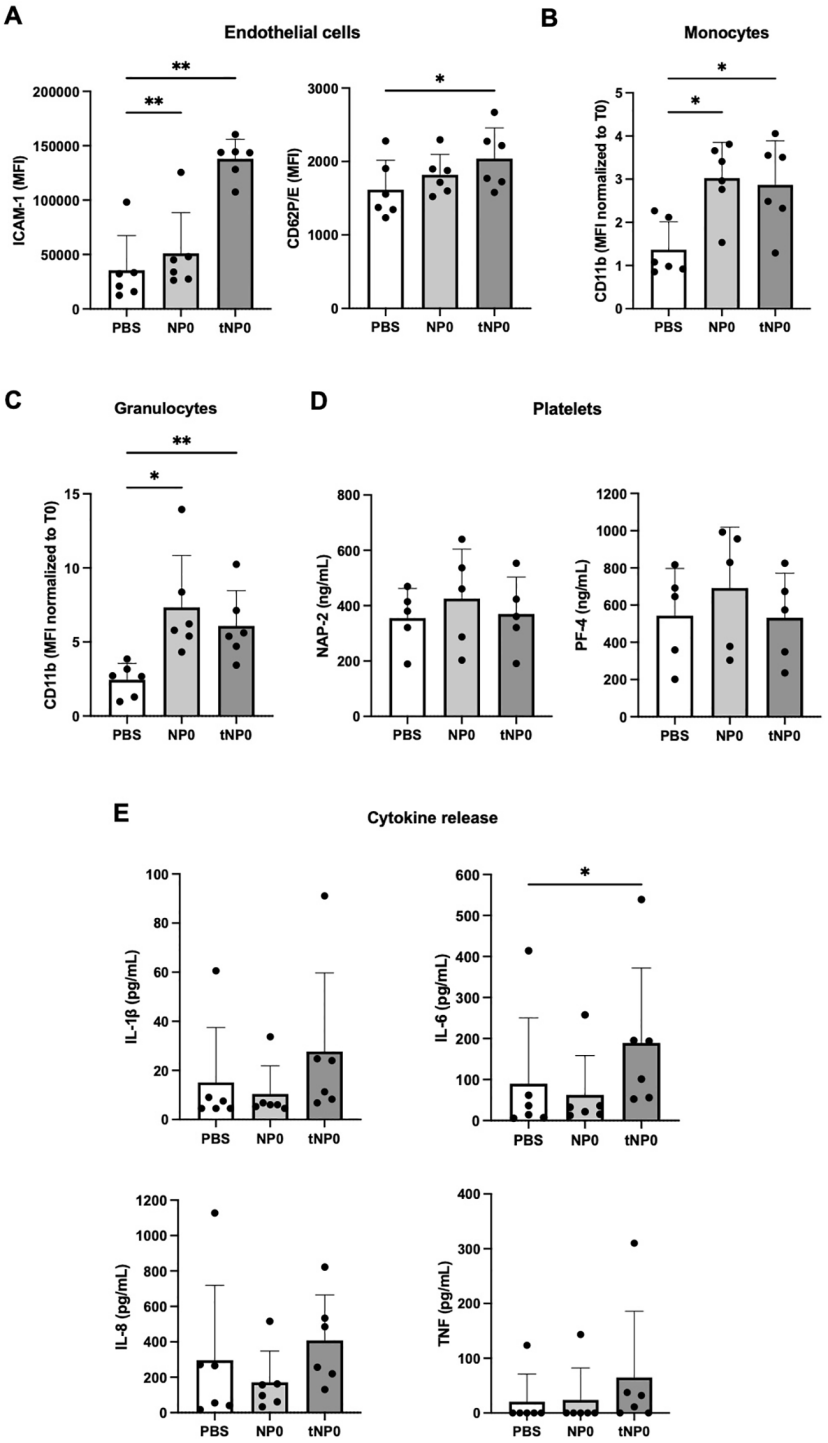
Endothelial cells and human blood cells activation. **(A)** HLMVECs endothelial cell activation evaluated by measuring ICAM-1 (left) and CD62P/E (right) expression by flow cytometry. Cell surface activation marker CD11b was measured on monocytes **(B)** and granulocytes **(C)** by flow cytometry. Platelets activation **(D)** was evaluated in plasma by ELISA, with the analysis of soluble markers NAP-1 (left) and PF-4 (right). **(E)** Cytokine release in plasma was analyzed with a 4-plex cytokine Luminex assay for IL-1β (upper left), IL-6 (upper right), IL-8 (lower left) and TNF (lower right). Sample treated with PBS as negative control. The values are shown as mean ± SD of n=6. Statistical was calculated by One-way ANOVA multiple comparison test. * = P ≤ 0.05; ** = P ≤ 0.01.

In order to evaluate the potential toxicity of NPs, each formulation was subjected to *in vitro* testing. BJAB and JURKAT cells were selected as relevant CD19^+^ and CD19^-^ cell models, respectively. The interaction between cells and NPs did not result in cell death after 24 hours of incubation. In fact, it was observed that the mitochondrial activity of the cells, expressed as a percentage, increased in both cell lines in comparison to the negative control of cells treated with PBS, as determined by the MTT assay (**Figure 2A**). The capacity of NPs to lyse red blood cells was evaluated through the assessment of hemoglobin release as a product of direct hemolysis. No evidence of mechanical disruption of erythrocytes was observed (**Figure 2B**). A turbidity assay was conducted to investigate whether NPs interfere with the coagulation process (**Figure 1C**); NPs were incubated with NHP, and a solution containing Ca^2+^ was added to initiate the coagulation process. The turbidity, resulting from the activation of the coagulation process and clot formation, was analyzed over time, demonstrating that the coagulation exhibited a similar trend in the presence or absence of NPs. The half-coagulation time was extrapolated, and the data demonstrated that there was no statistically significant difference between the sample treated with NP0 and the positive control (CTRL^+^), which was represented by non-treated plasma. In contrast, the presence of tNP0 resulted in a significant reduction in the half-coagulation time in comparison to the CTRL^+^. The interaction with the complement system was investigated via a CH50 hemolytic assay (**Figure 2D**), which expressed the residual activity of the classical pathway, from initiation to its conclusion. The NHS was incubated with both NPs and PBS, which served as the positive control (CTRL^+^). The number of hemolytic units per mL was found to be similar between all conditions for both NP0 and tNP0, indicating that NPs did not significantly impair complement system function. To further investigate the impact of NPs on the innate immune response, a human whole blood with endothelial cells model was employed. To this end, lepirudin-anticoagulated human blood from 6 different healthy human donors was co-incubated with HLMVECs with or without NPs, for up to 4 hours. To evaluate whether NPs affected complement system activation, the complement C3-activation markers C3b and C3c, and the soluble terminal complement sC5b-9 complex, were quantified by ELISA (**Figure 2E**). The results demonstrated that, in comparison to whole blood treated with PBS, which served as the negative control, NPs significantly enhanced complement system activation, independently of the presence of the targeting mechanism. The inflammatory response induced by the interaction between NPs, HLMVECs, and whole blood was investigated through the quantification of cell surface inflammation markers and cytokine release, employing flow cytometry and ELISA, respectively. As illustrated in **Figures 3A–D**, the data obtained from flow cytometry revealed that following exposure to NPs, HLMVECs endothelial cells, and blood cells (specifically monocytes and granulocytes) exhibited an activation, as evidenced by an increase in the MFI compared to the PBS treatment, which served as the negative control. In particular, HLMVECs in blood exhibited a significantly higher expression of Intracellular Adhesion Molecule 1 (ICAM-1) (**Figure 3A**) after exposure to both NP0 and tNP0 compared to the negative control. Additionally, an increased expression of CD62P/E Selectin (**Figure 3A**) was observed only in samples treated with tNP0 in contrast to those treated with PBS. Monocytes and granulocytes were isolated from the whole blood sample after the initial 15 minutes of incubation due to the rapid activation of these cells. To account for the considerable inter-donor variability in the response, the MFI values were normalized to the basal levels observed at time point T0 prior to exposure to NPs. The cell surface activation marker CD11b was observed to be significantly highly expressed on the cell surface of both monocytes (**Figure 3B**) and granulocytes (**Figure 3C**) in comparison to samples treated with PBS. Platelets are cells with an important role in the thrombo-inflammatory response and it was evaluated whether NPs affect their activation in whole blood. The soluble activation markers NAP-2 and PF-4 (**Figure 3D**) were analyzed in plasma isolated from the whole blood after incubation for 4 hours. No significant activation was observed, as evidenced by comparable levels following incubation with both NPs and PBS. Given the observed activation of endothelial cells, monocytes, and granulocytes was observed, the release of IL-1β, IL-6, IL-8, and TNF was quantified in plasma. As illustrated in **Figure 3E**, despite the activation of the aforementioned cited cell types, the cytokine release was comparable to that induced by PBS. Only an IL-6 release was discernible following exposure to tNP0. Furthermore, variations in cell blood count and hemoglobin were considered (**Supplementary Table 1**). The number of red and white blood cells and hemoglobin levels in samples treated with NPs remained unaltered, indicating that erythrocyte lysis did not occur. Nevertheless, a slight reduction was noted in platelet numbers in comparison to samples treated with PBS.

### 3.3 *In vitro* evaluation of the activity of the targeting mechanism

**Figure 4:**
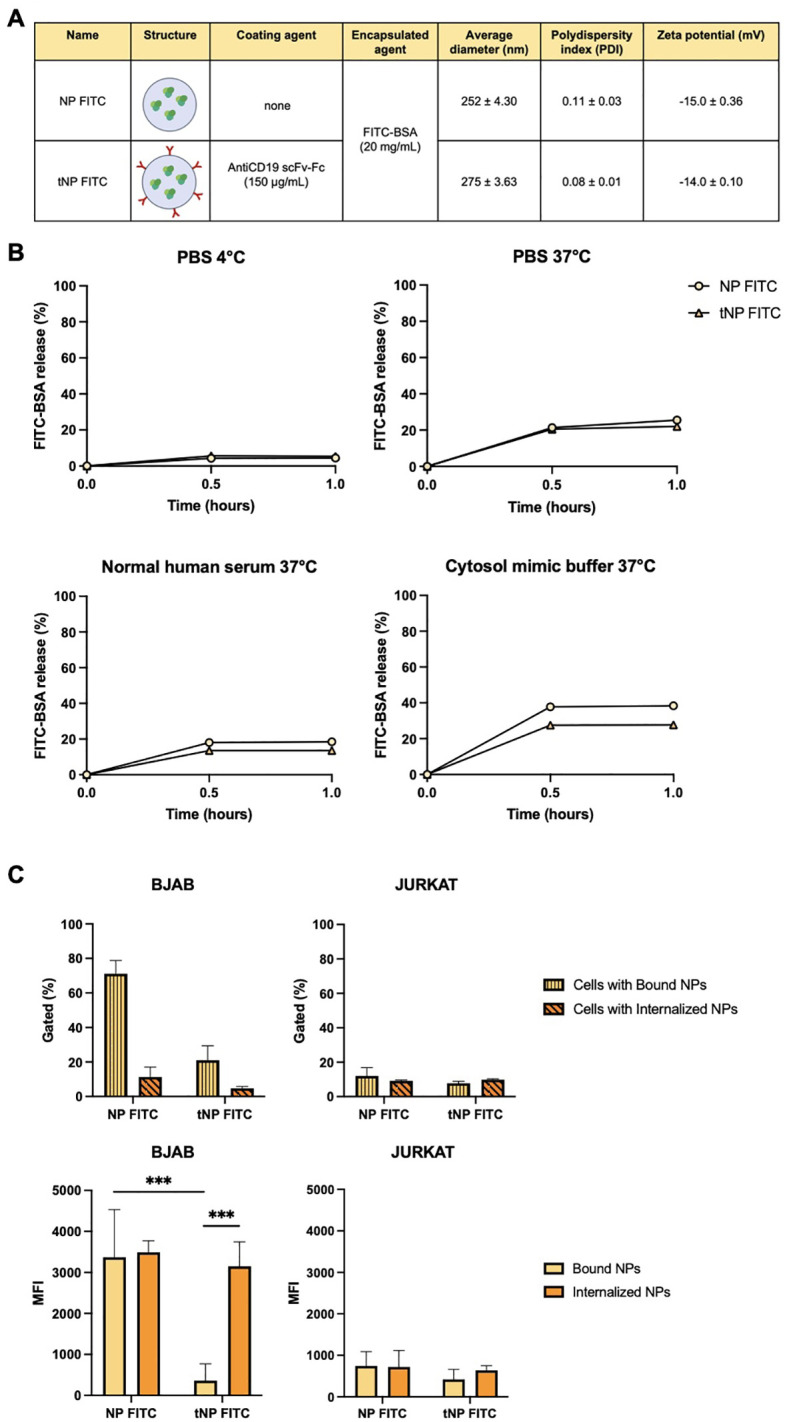
*In vitro* effectiveness of the antiCD19 targeting system. **(A)** Summary of the composition and features of NP FITC and tNP FITC obtained by DLS. **(B)** Release studies of FITC-BSA from NPs in PBS at 4°C (upper left), PBS at 37°C (upper right), diluted normal human serum at 37°C (lower left) or cytosol mimic buffer at 37°C (lower right) for 0.5-1 hour. The values are shown as mean ± SD of n=2. **(C)** Binding/internalization studies of NPs performed for 1 hour on BJAB (left panels) and JURKAT cells (right panels) by flow cytometry. Data are expressed as % of cells interacting with NPs (upper panels) and the amount of NPs (MFI) interacting with cells (lower panels). Values are shown as mean ± SD of n=3. Statistical was calculated by One-way ANOVA multiple comparison test. *** = P ≤ 0.001.

Flow cytometry and fluorescence microscopy techniques were exploited to evaluate whether the presence of the targeting mechanism ensured a selective interaction with CD19^+^ target cells. Two novel formulations were synthesized resulting in NP FITC (untargeted) and tNP FITC (targeted). Both formulations consisted of an aqueous inner core composed of FITC-BSA. The different payload did not affect the physicochemical parameters, which are summarized in **Figure 4A**, nor did it influence the antiCD19 coupling efficiency. The encapsulation efficiency of FITC-BSA was estimated by an indirect method, whereby the difference between the initial amount of FITC-BSA added during the preparation process and the remaining amount in the aqueous suspension after centrifugation and washing steps was measured by employing the fluorescent properties of FITC. The fluorescent values obtained were interpolated with a FITC-BSA standard curve, and the amount of encapsulation was extrapolated. This yielded a value of approximately 70% for both preparations, which lends support to the reproducibility of the synthesis process. Furthermore, the fluorescent signal of both NPs was quantified immediately following synthesis, designated as T0. This confirmed the homogeneity of the two formulations, which exhibited comparable fluorescence values (**Supplementary Figure S1A**, left). The spontaneous payload release was evaluated over a one-year period at storage conditions of 4°C by quantifying the fluorescence signal of NPs. As illustrated in **Supplementary Figure S1A** (right), both formulations exhibited a slight release of FITC-BSA, resulting in slightly lower values of fluorescence detected in comparison to that detected at T0. This confirms their stability and suitability for use beyond one year. To further characterize the structure of NPs, release studies were conducted in a variety of buffers and conditions for up to one hour (**Figure 4B**), which represents the incubation time for binding/internalization studies, described in detail in the subsequent paragraph. In storage conditions (PBS at 4°C) the payload release was observed to be lower than 10% and comparable between the two formulations. At 37°C and pH 7.4, both FITC-BSA NPs exhibited a burst release of approximately 20% of the payload within 0.5 hours, followed by a plateau. A comparable pattern was observed in diluted NHS, which simulated the presence of all serum proteins. The main release was observed to be approximately 40% in a simulated cytosol (cytosol mimic buffer), which prompted an assessment of fluorescence stability following cell internalization.

The interaction between NPs and cells, as well as the efficacy of the targeting system, were investigated by flow cytometry on both BJAB and JURKAT cells that were incubated with NP FITC or tNP FITC for 1 hour at 37°C (**Figure 4C**). BJAB cells exhibited greater interaction when incubated with NP FITC. In fact, the percentage of gated cells with bound NP FITC is higher compared to tNP FITC (~70% versus ~20% respectively; **Figure 4C**, upper left). To further investigate this aspect, we focused on the pH-dependent fluorescence properties of FITC-BSA (**Supplementary Figure S1B**, left). To this end, FITC-BSA was resuspended in a series of buffered solutions with varying pH values (between 4.0 and 8.0), and the fluorescent signal was calculated as previously described. The CTCF of FITC-BSA, expressed as a percentage, exhibited a pH-dependent fluorescence profile, with higher values observed at alkaline-neutral pH and lower values at acidic pH. This resulted in a decrease of approximately 30% in fluorescence intensity. This demonstrated the pH sensitivity of FITC, and the same trend was observed for NPs encapsulating FITC-BSA (**Supplementary Figure S1B**, right). To detect only cells that had internalized NPs, BJAB were treated with Pronase, a protease mixture. The proteolytic activity of Pronase results in the degradation of surface proteins that are responsible for mediating the attachment of NPs to the cell. This process disrupts both specific (ligand-receptor) and non-specific interactions, resulting in the release of externally bound NPs. NPs that are internalized and protected within the cell remain intact, as the Pronase enzymes are unable to access them. Following treatment, approximately 10% of BJAB treated with NP FITC and ~7% of BJAB treated with tNP FITC exhibited internalized NPs (**Figure 4C**, upper left). Instead, the MFI was employed to estimate the rate of NPs internalization. BJAB cells (**Figure 4C**, lower left) exhibited a comparable amount of bound and internalized NP FITC (MFI of 3400 versus 3500). In contrast, the signal associated with tNP FITC interacting with BJAB indicated that the majority of NPs were internalized within cells. The MFI values of bound tNP FITC in BJAB were significantly lower in comparison to NP FITC (360 versus 3400). In fact, the MFI values of internalized tNP FITC were significantly higher than those of the bound NPs (3150 versus 360). In JURKAT cells, no significant differences were observed in the percentage of gated cells with bound NPs for both types of NPs (**Figure 4C**, upper right). The percentage of cells with bound NPs was found to be approximately 12% for NP FITC and approximately 7% for tNP FITC. Following treatment with Pronase, this resulted in approximately 9% for both formulations. The relevance of the targeting mechanism was confirmed by analyzing the MFI related to the number of interacting NPs. In this instance, the values of the bound or internalized NPs were comparable for both formulations. The MFI for bound NPs is approximately 740 for NP FITC and approximately 420 for tNP FITC while the MFI for internalized NPs is approximately 720 and 640, respectively (**Figure 4C**, lower right).

### 3.4 *In vivo* evaluation of the activity of the targeting mechanism

**Figure 5:**
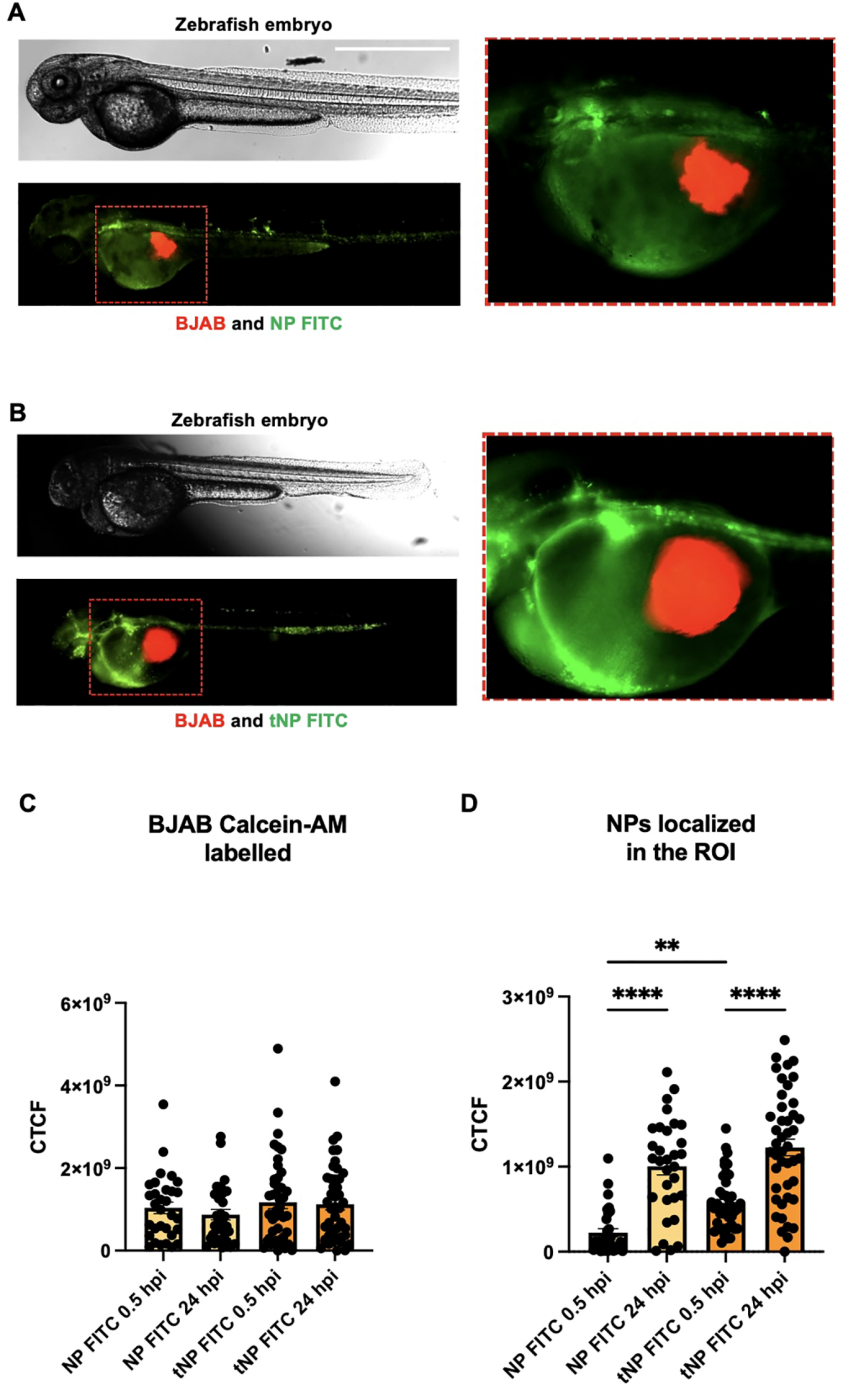
NPs biodistribution studies in a human/zebrafish lymphoma xenograft model. Representative images at 0.5 hours post injection (hpi) of wild type zebrafish embryos (upper panels) injected with Calcein-AM labeled BJAB (red fluorescence signal) in the perivitelline space and NP FITC **(A)** or tNP FITC **(B)** in the duct of Cuvier (green fluorescence signal, lower panels). Region Of Interest (ROI) chosen for the analysis of the fluorescence signals represented in the left panels (Scale bar 1000 μm) **(C)** Data analysis of the CTCF of the red-fluorescent areas (BJAB cells) in the perivitelline space of zebrafish embryos injected with NP FITC or tNP FITC at 0.5 and 24 hpi. **(D)** Data analysis of the CTCF of the green-fluorescent areas (NPs) which arrives in the yolk of zebrafish embryos at 0.5 and 24 hpi. The values are shown as mean ± SEM of n=40. Statistical was calculated by One-way ANOVA multiple comparison test. ** = P ≤ 0.01; **** = P ≤ 0.0001.

The role of the targeting mechanism was evaluated in a human/zebrafish xenograft model of B-cell lymphoma. A localized tumor-bearing zebrafish model was initially established by injecting approximately 2,500 Calcein AM-labeled BJAB cells into the perivitelline space of embryos at 48 hpf. Subsequently, FITC-BSA NPs were then injected into the duct of Cuvier and the embryos were observed under fluorescence microscopy for the subsequent 24 hours (**Figures 5A, B**). Following the injection, no indications of toxicity (e.g., pericardial enlargement and alterations in pigmentation) resulting from NPs or BJAB cells were observed for up to 72 hpi. The interaction between cancer cells in the perivitelline space and NPs injected in the circulation was observed by delineating a ROI on the tumor area in the perivitelline space. As illustrated in **Figure 5C**, the red fluorescence signal, expressed as CTCF, regarding the quantity of cells injected into the group of animals treated with NP FITC was comparable to that treated with the tNP FITC. This indicated that the animals received the same quantity of cells and that the model was reproducible. Furthermore, the animals did not significantly eliminate the cells, and the cell fluorescence signal after 24 hours was comparable to that detected at 0.5 hours. NPs from the bloodstream exhibited differential accumulation into the perivitelline space based on the presence of the antiCD19 (**Figure 5D**). At 0.5 hpi, the green fluorescence signal in the perivitelline space demonstrated significantly elevated values for tNP FITC compared to NP FITC, substantiating the relevance of the targeting mechanism in the interaction with cells *in vivo* as well as *in vitro*. At 24 hpi, both NP FITC and tNP FITC demonstrated accumulation in the tumor region. Notably, the green fluorescent signal reached values significantly higher than those observed at 0.5 hpi. However, longer evident at 24 hpi, when both types of NPs had accumulated to a similar extent. Similarly, the absence of a significant difference in the fluorescent signal between NP FITC and tNP FITC was corroborated by the decrease in FITC fluorescence associated with pH-dependent internalization following higher internalization in target cells, both *in vitro* and *in vivo*.

### 3.5 The targeting mechanism promotes transfection of tumor B cells

**Figure 6:**
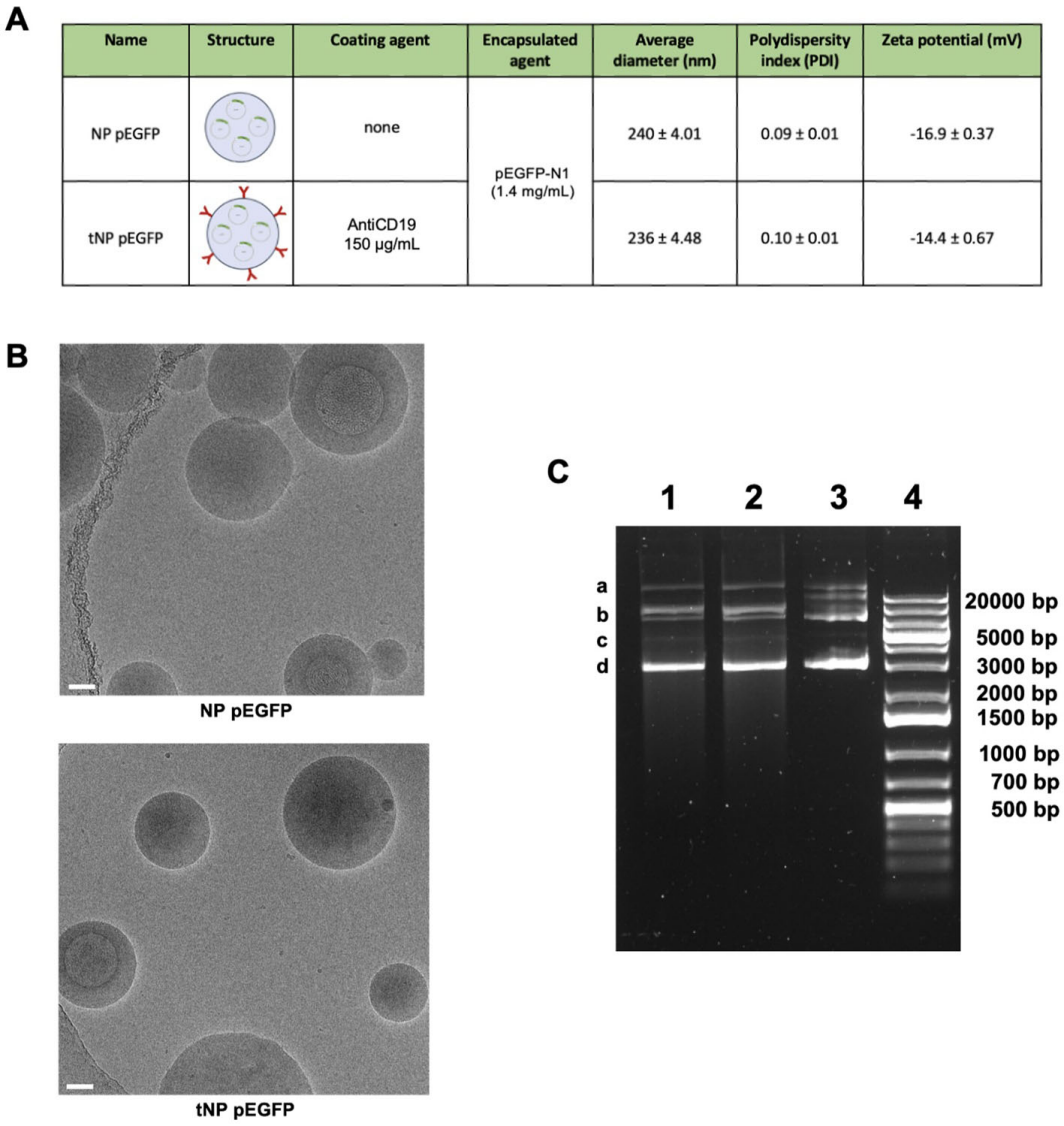
Characterization of DNA-loaded NPs. **(A)** Summary of the composition and features of NP pEGFP and tNP pEGFP obtained by DLS. **(B)** Cryo-EM images of NP pEGFP and tNP pEGFP (scale bar 50 nm). **(C)** Agarose gel electrophoresis of NP pEGFP (Lane 1), tNP pEGFP (Lane 2) and purified pEGFP-N1 (Lane 3) in comparison to molecular weight (Lane 4). pEGFP-N1 shows different conformations: open circular (a), relaxed (b), linear (c), and supercoiled (d).

In order to establish this platform available for exogenous protein production, pDNA-loaded NPs were produced by encapsulating the EGFP-coding plasmid pEGFP-N1 in the aqueous core of NPs. This resulted in two new formulations, untargeted (NP pEGFP) and targeted (tNP pEGFP). With regard to FITC-BSA NPs, the different payload did not affect the size, PDI, and charge parameters, as illustrated in **Figure 6A**. Furthermore, no impact was observed on antiCD19 coupling efficiency. It is evident that, despite the pDNA negativity, the zeta potential of both preparations remains comparable to that of the aforementioned formulations. The pDNA release from NPs was analyzed in a manner analogous to that employed for FITC-BSA NPs in the previously described release buffers. These findings demonstrated that the amount of pDNA released from NPs was comparable to that of FITC-BSA, and that the pDNA remained intact even in a cytosol mimic buffer. This was a crucial step in enabling cell transfection. To gain insight into the structural characteristics of the NPs, cryo-EM technology was employed to construct models for detailed analysis. **Figure 6B** illustrates a densely packed and disordered core of nucleic acid. The quantity of pDNA incorporated into NPs was determined through an indirect approach, whereby the difference between the total amount of pDNA introduced during the preparation step and the residual pDNA in the solution following NPs formation was calculated. The results demonstrated that the encapsulation efficiency is comparable to that of FITC-BSA NPs. The structural integrity of the encapsulated pDNA was evaluated by agarose gel electrophoresis after extraction of the payload from the core of NPs (**Figure 6C**). The analysis confirmed the substantial integrity of pDNA despite the synthesis process being the extracted pDNA conformation comparable to the untreated control.

**Figure 7:**
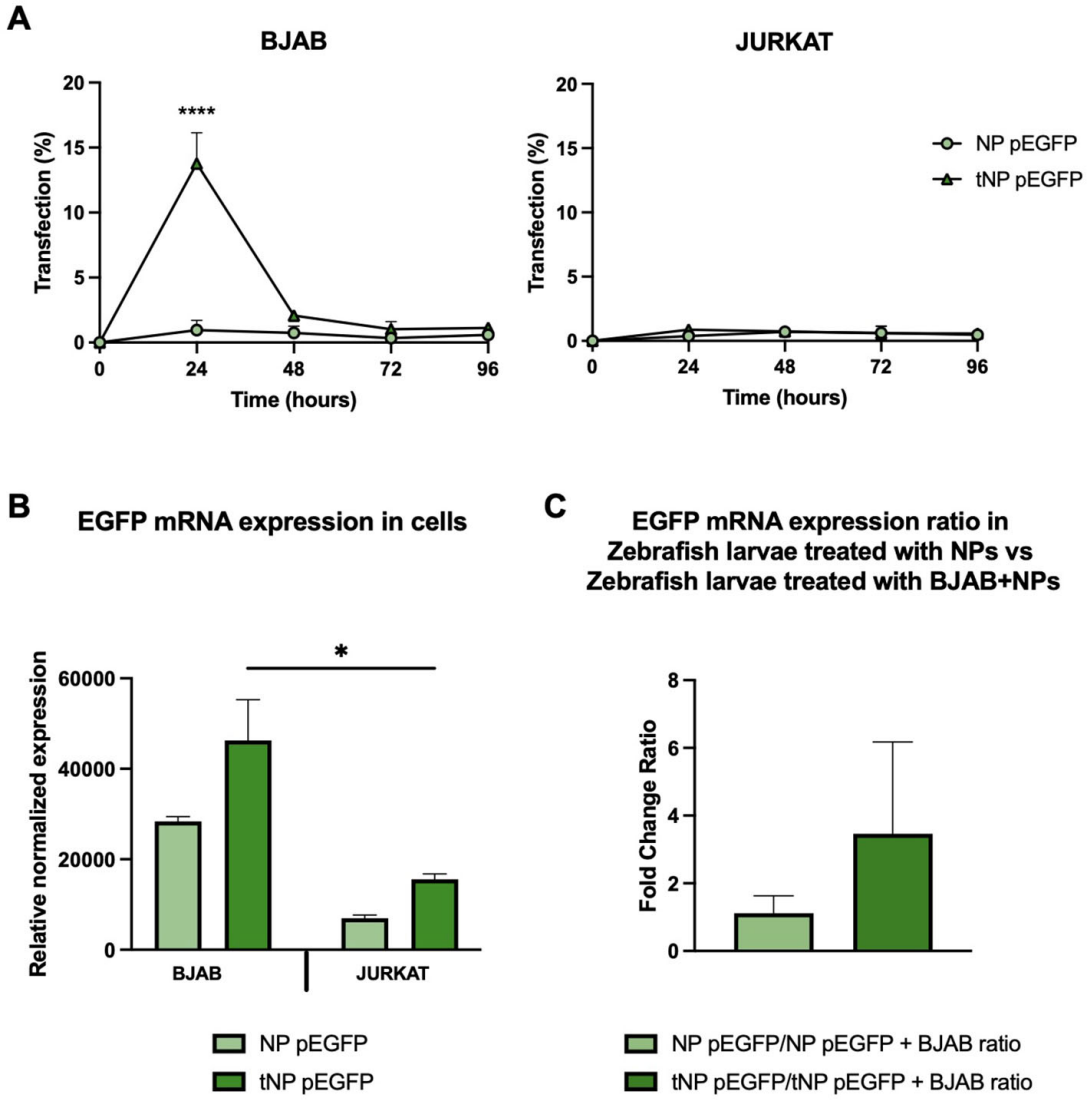
*In vitro* and *in vivo* evaluation of the transfection efficiency. **(A)** Transfection studies of NPs performed for up 96 hours on BJAB (left panels) and JURKAT cells (right panels) by flow cytometry. Data are expressed as % of cells expressing EGFP. The values are shown as mean ± SD of n=3. Statistical was calculated by One-way ANOVA multiple comparison test. **** = P ≤ 0.0001. **(B)** qRT-PCR expression analysis of EGFP in BJAB and JURKAT cells at 24 hours post transfection with NP pEGFP and tNP pEGFP. Expression data are normalized against human *RPL34* gene. The values are shown as mean ± SD of n=3. Statistical was calculated by One-way ANOVA multiple comparison test. * = P ≤ 0.05 **(C)** qRT-PCR expression analysis of EGFP in the human/zebrafish lymphoma xenograft model at 24 hpi. Expression data are normalized against zebrafish *β-actin* gene and expressed as ratio between samples treated with only NPs and samples treated with both BJAB cells and NPs. The values are shown as mean ± SD of n=3. Statistical was calculated by unpaired t-test.

The efficacy of this pDNA delivery platform was evaluated through *in vitro* transfection studies in both BJAB and JURKAT cells, to elucidate the role of the targeting mechanism (**Figure 7A**). Following a 24-hour incubation period with a volume of NPs equivalent to 3 μg of encapsulated pDNA, any remaining NPs that had not interacted with cells were removed and replaced with fresh medium. The expression of EGFP was evaluated for up to 96 hours by flow cytometry. The results, expressed as a percentage of GFP^+^ cells, demonstrated that the transfection efficiency was dependent on the presence of the targeting mechanism, particularly antiCD19 for tumor B cells. In fact, at 24 hours post-transfection, approximately 15% of BJAB cells treated with tNP pEGFP exhibited expression of the fluorescent protein, in comparison to those treated with NP pEGFP, which exhibited a lower (~2%) transfection efficiency. The efficiency of tNP pEGFP-mediated transfection declines over time, reaching a level comparable to that of NP pEGFP at 96 hours post-transfection. In JURKAT cells, the absence of the CD19 antigen precludes the internalization of NPs, thereby preventing EGFP expression. Only approximately 2% of transfected cells expressed the fluorescent protein, regardless of whether NP pEGFP or tNP pEGFP were used. mRNA levels were employed as an additional indicator of EGFP gene expression. Consequently, cDNA was obtained from BJAB and JURKAT cells treated with the two pDNA-loaded NPs formulations for 24 hours, and a qRT-PCR was conducted (**Figure 7B**). The results were in accordance with the data obtained from flow cytometry, indicating that BJAB cells transfected with tNP pEGFP exhibited higher transcription levels of EGFP mRNA, as quantified by Relative Normalized Expression, than those treated with NP pEGFP. A comparable profile was discerned in JURKAT cells, though with diminished expression values relative to BJAB cells. These findings reinforce the importance of the targeting mechanism in enhancing the internalization of NPs in CD19^+^ cell lines, which leads to substantial EGFP transcription and expression. Subsequently, the transfection efficiency was evaluated *in vivo* in the human/zebrafish xenograft model of B-cell lymphoma (**Figure 7C**). Approximately 2,500 unlabeled BJAB were injected into the perivitelline space and NP pEGFP or tNP pEGFP (corresponding to 0.8 ng of encapsulated pDNA) were injected into the duct of Cuvier 48 hpf. cDNA was obtained from whole embryos at 24 hpi and samples of embryos treated with only NPs were compared to those treated with BJAB cells and NPs to assess whether the presence of human CD19^+^ cells could enhance the protein expression in the embryos. The results demonstrated that the fold change ratio between samples treated with only tNP pEGFP and samples treated with BJAB cells and tNP pEGFP is approximately 3 times higher than the ratio between samples treated with only NP pEGFP versus samples treated with BJAB cells and NP pEGFP. This evidence corroborates the conclusion that, even in a more complex model, the targeted NPs exhibit superior performance in terms of mediating transfection when compared to their untargeted counterparts in the presence of a CD19^+^ system.

## 4 Discussion

The use of non-viral approaches, such as NPs, for the regulation or expression of genes has been investigated for potential clinical applications, including the development of vaccines and gene editing techniques. Examples of FDA-approved products that utilize this technology include the BioNTech/Pfizer and Moderna mRNA vaccines for the treatment of SARS-CoV-2 infection, as well as Onpattro, a siRNA formulation for the treatment of hereditary transthyretin amyloidosis (hATTR) (31). In this context, RNA has emerged as a highly useful therapeutic agent, requiring action in the cytoplasm, and thus, also effective in non-dividing cells (32). RNA-based therapy presents a promising avenue for treatment, yet it is not without its limitations. One challenge is the inherent instability of RNA, which can be susceptible to degradation by ubiquitous RNases. Additionally, the storage of RNA-based therapies presents a significant hurdle (33). To address these issues, double-stranded DNAs (e.g., pDNA) represent a more stable alternative, particularly given their preferential action in proliferating cells (32).

Based on these considerations, our work demonstrates that the presence of an antiCD19 targeting mechanism on the surface of PLGA-PVA NPs enhances the internalization of NPs and subsequent transfection of tumoral B cells while maintaining a favorable safety profile.

NPs can be produced with a scalable and reproducible process and demonstrated physical stability for up to 1 year. NPs were spherical in shape, negatively charged, and smaller than 300 nm, irrespective of the payload. In the literature, several cationic NPs are described, and they are associated with a high general cellular uptake (12, 34, 35). This is supposedly due to favorable electrostatic interactions, which may result in higher toxicity and lack of specificity. Conversely, the negative charge of these PLGA-PVA NPs constrains the occurrence of these unfavorable interactions, necessitating the use of a targeting mechanism to facilitate a selective NP-cell interaction. The pivotal function of NPs diameter in the clearance, circulation time, and internalization is well-established. NPs of a high size result in a low internalization rate (36, 37) and a main accumulation in the liver (38). The orientation of the antiCD19 targeting mechanism and the accessibility of the variable regions for target binding were confirmed, as previously demonstrated by our laboratory for scFv-Fc constructs with a similar design (39).

The ability of PLGA-PVA NPs to encapsulate and retain payloads was demonstrated. The release of encapsulated materials under physiological conditions exhibited an initial burst release, which was visible within 0.5 hours. This phenomenon is associated with the physicochemical characteristics of polymeric NPs (40). The data obtained from the release in cytosol mimicking buffer were of particular significance, as they demonstrated the persistence of the payload features even after the NPs had been internalized.

FITC-BSA loaded NPs were employed to monitor the interaction between NPs and cells, as well as the internalization process. PLGA NPs are primarily internalized via clathrin-mediated endocytosis (41), a process that begins with the formation of an endocytic vesicle and progresses to an early endosomal compartment, eventually maturing into a late endosome (42). The late endosome subsequently fuses with a lysosome, where NPs and their cargo can be degraded. It is established that the CD19 antigen undergoes internalization following binding with antibodies (43). Consequently, the interaction between the antiCD19 targeting mechanism on NPs and the CD19 antigen on the cell surface can facilitate an increased internalization rate. The targeting mechanism did not appear to enhance interaction with CD19^+^ cells. However, the pH-dependent properties of FITC may explain these findings. The acidic environment can result in the proteolytic degradation of FITC-BSA (44) and a notable reduction in the fluorescence of FITC-BSA NPs, which in turn leads to a decline in MFI and a diminished percentage of positive CD19^+^ cells. In fact, mostly NP FITC remained bound to the cell surface of CD19^+^, with minimal internalization, thereby preserving their fluorescence. This resulted in a higher percentage of positive cells detected. On the contrary, tNP FITC were more internalized, but most of the fluorescent signal was probably lost due to the pH-dependent properties of FITC-BSA. The results from CD19^-^ cells corroborate this hypothesis, demonstrating no difference between NP FITC and tNP FITC. This indicates that both types of NPs remained predominantly on the cell surface and retained their fluorescence. In an *in vivo* human lymphoma model in zebrafish, the injection of tNP FITC into the bloodstream resulted in a more localized distribution in proximity to the tumor mass after a short period of 0.5 hours. This observation highlights the significance of the targeting mechanism for achieving effective interaction between tumors and NPs injected into the bloodstream, aligning with previous findings from our group in a comparable model (45). As with the *in vitro* studies, the pH-related decrease in FITC fluorescence may explain the lack of a significant difference between NP FITC and tNP FITC in the accumulation in the perivitelline space over time. In fact, similar results were obtained from the analysis conducted at 0.5 and 24 hpi. The signal from NP FITC and tNP FITC increased from 0.5 to 24 hours, reaching comparable values between the two formulations at 24 hours. As with the *in vitro* studies, it is postulated that tNP FITC were more internalized in the tumor mass, resulting in a reduction in the fluorescent signal due to the pH-dependent properties of FITC-BSA. Altogether these data demonstrated that targeted NPs improved the delivery of loaded material into CD19^+^ cells, in both *in vitro* and *in vivo* settings, and the interaction between the targeting mechanism and the CD19 antigen is shown to contribute to NPs internalization. As previously documented in the literature (46) regarding other types of NPs, it has been observed that zebrafish embryos frequently exhibit morphological alterations suggestive of inflammatory processes, including pericardial enlargement and alterations in pigmentation. The presence of these alterations was evaluated through microscopic examination, thereby providing an initial indication of the presence of inflammation or toxicity. No alterations were detected, which may be attributed to the reduced production and accumulation of inflammatory mediators *in vivo* in the zebrafish model, in comparison to their accumulation and subsequent inflammatory effects observed *in vitro*.

The delivery of the EGFP-coding plasmid demonstrated the expression of both specific mRNA and protein mostly in CD19^+^ cells. The highest transfection efficiency occurs in CD19^+^ cells, with low values for the CD19^-^. However, the mRNA detected in CD19^-^ cells demonstrated that a quote of NPs, targeted or not, enter the cells. This discrepancy could be attributed to a nonspecific interaction between the cell membrane and NPs, which can be nonspecifically internalized by macropinocytosis (47). In addition, this can explain the internalization of FITC-BSA loaded NPs which was also observed. In the human/zebrafish xenograft lymphoma model, the ratio of EGFP mRNA levels in animals treated with only tNP and animals treated with tNP and CD19^+^ cells was 3 fold higher than that of animals treated with only NP and animals treated with NP and CD19^+^ cells. This supports that in a more complex system, tNP ensured a higher EGFP expression, probably due to the internalization in the CD19^+^ cells present, even though it was not possible to establish whether the EGFP expression was only in CD19^+^ cells injected and not in zebrafish cells. It is essential to acknowledge the value of the human/zebrafish xenograft lymphoma model as a preliminary research tool, offering significant insights due to its optical transparency and rapid development. This model provides a foundation for understanding the biodistribution and targeting mechanism efficiency of NPs *in vivo*. However, it is important to recognize the limitations of this model, including an underdeveloped immune system, a restricted experimental window for the use of embryos (5 days post-fertilization), and differences in organ maturation and metabolic pathways, which limit the information available about NPs clearance (48, 49).

Particular attention was given to the safety of this approach. *In vitro* studies have demonstrated that NPs do not induce cytotoxicity in a range of cell lines. Instead, they appear to enhance cell viability, as evidenced by elevated mitochondrial activity in MTT assays. PLGA is noted to be biodegradable and biocompatible, and its internalization and degradation produce molecules that fuel the Krebs cycle (50–52). *In vitro* models demonstrated that NPs failed to lyse red blood cells or to strongly interfere with the hemostatic system or the complement system. These were relevant features based on their properties, including size, shape, and surface properties. Their assessment was imperative as NPs introduced into the circulatory system can interact with serum proteins, forming a coating layer known as the protein corona, which may potentially elicit an inflammatory response (22, 53). Our previous experiments demonstrated the deposition of antibodies and proteins of the coagulation and complement systems on the surface of PLGA-PVA NPs incubated with human serum (25). The data on NPs behavior in an *ex vivo* whole blood model combined with endothelial cells was included in an effort to mimic the response in a blood vessel. By using antibodies recognizing activation-specific neoepitopes in activated C3, i.e., C3b, iC3b and C3c, and C9 when incorporated in the C5b-9-complex (28, 54), complement activation induced by the NPs was confirmed. In consideration of these findings, it is postulated that the potential deposition of C3b on the surface of NPs may facilitate their recognition and uptake by macrophages via complement receptors, thereby accelerating their clearance and potentially influencing therapeutic efficacy.

The activation of endothelial cells, which play a pivotal role in regulating blood vessel function, was evaluated by examining the expression of ICAM-1 and CD62P/E. These molecules are upregulated upon endothelial cell activation as part of the inflammatory response. The expression of these adhesion molecules was notably elevated in the presence of tNP. ICAM-1 and CD62P/E are expressed in response to complement activation fragments and proinflammatory cytokines, including IL-1β and TNF. The levels of these cytokines, as well as IL-6 and IL-8, were particularly low, thereby inducing the focus of endothelial cell activation to C5a and its interaction with C5a receptors (55–58). Complement activation products have been demonstrated to activate monocytes, granulocytes, and platelets, particularly via the C5a-C5aR1 axis (59). Monocytes and granulocytes exhibited only minimal activation, as evidenced by the expression of surface adhesion molecules and the absence of inflammatory cytokines that are typically produced following their activation (60).

The integrity of the cells was considered to be an indicator of biocompatibility, and no hemoglobin, a byproduct of hemolysis, was released by the cells. In a whole blood model combined with endothelial cells, there was no effect on blood cell numbers, red blood cell heterogeneity, or average size, indicating a limited nonspecific interaction with NPs.

These *in vitro* data, in conjunction with the absence of toxicity in the human/zebrafish xenograft lymphoma model, suggest a favorable safety profile of these NPs, although further studies are required to fully assess their long-term safety and *in vivo* effects in more complex models. The results obtained support the use of antiCD19 targeted NPs as a promising approach for gene delivery to preferentially transfect CD19^+^ target cells. This platform demonstrates the potential for the production of therapeutic proteins, including suicide or cytotoxic proteins and antibodies, within the tumor microenvironment. This approach may facilitate more effective outcomes while minimizing the occurrence of significant side effects.

## 5 Conclusion

PLGA-PVA NPs demonstrated a favorable safety profile both *in vitro* and *in vivo*, highlighting their biocompatibility and suitability for biomedical applications. The incorporation of a targeting mechanism enhanced the interaction with target cells, suggesting improved cellular uptake. The findings support the application of this nanoplatform in gene therapy, as a DNA delivery system for the local expression of therapeutic proteins.

## Data Availability

The raw data supporting the conclusions of this article will be made available by the authors, without undue reservation.
