# Supplementary material for: DNA-loaded targeted nanoparticles as a safe platform to produce exogenous proteins in tumor B cells
Source: Front Immunol. 2025 Jan 22;15:1509322. doi: 10.3389/fimmu.2024.1509322 (PMC11794205; doi:10.3389/fimmu.2024.1509322)
Supplement: Supplementary file 1 [file Table1.docx]

Supplementary Material

| PBS | NP0 | tNP0 |
| --- | --- | --- |

| **RBC (10^12^/L)** | 4.65 ± 0.83 | 4.62 ± 0.66 | 4.54 ± 0.74 |
| --- | --- | --- | --- |
| **PLT (10^9^/L)** | 152.40 ± 30.25 | 149.2 ± 18.55 | 146.8 ± 13.16 |
| **WBC (10^9^/L)** | 4.16 ± 0.75 | 4.38 ± 0.93 | 4.12 ± 0.85 |
| **LYM (10^9^/L)** | 1.48 ± 0.33 | 1.42 ± 0.44 | 1.38 ± 0.39 |
| **GRN (10^9^/L)** | 2.28 ± 0.6 | 2.76 ± 0.72 | 2.56 ± 0.5 |
| **HGB (g/dL)** | 14.1 ± 2.53 | 14.16 ± 2.05 | 14.02 ± 2.23 |

**Table 1. Variation in blood cell count after incubation with PBS, NP0, tNP0.**

| **Primer name** | **Sequence 5’-3’** |
| --- | --- |
| **Zebrafish Beta actin Forward** | **CGAGCTGTCTTCCCATCCA** |
| **Zebrafish Beta actin Reverse** | **TCACCAACGTAGCTGTCTTTCTG** |
| **EGFP Foward** | **AGAACGGCATCAAGGTGAAC** |
| **EGFP Reverse** | **TGCTCAGGTAGTGGTTGTCG** |
| **L34 Forward** | **GTCCCGAACCCCTGGTAATAGA** |
| **L34 Reverse** | **GGCCCTGCTGACATGTTTCTT** |

**Table 2. List of primers used for the qRT-PCR experiment.**
