# Supplementary material for: DNA-loaded targeted nanoparticles as a safe platform to produce exogenous proteins in tumor B cells
Source: Front Immunol. 2025 Jan 22;15:1509322. doi: 10.3389/fimmu.2024.1509322 (PMC11794205; doi:10.3389/fimmu.2024.1509322)
Supplement: Supplementary file 2 [file Image1.pdf]

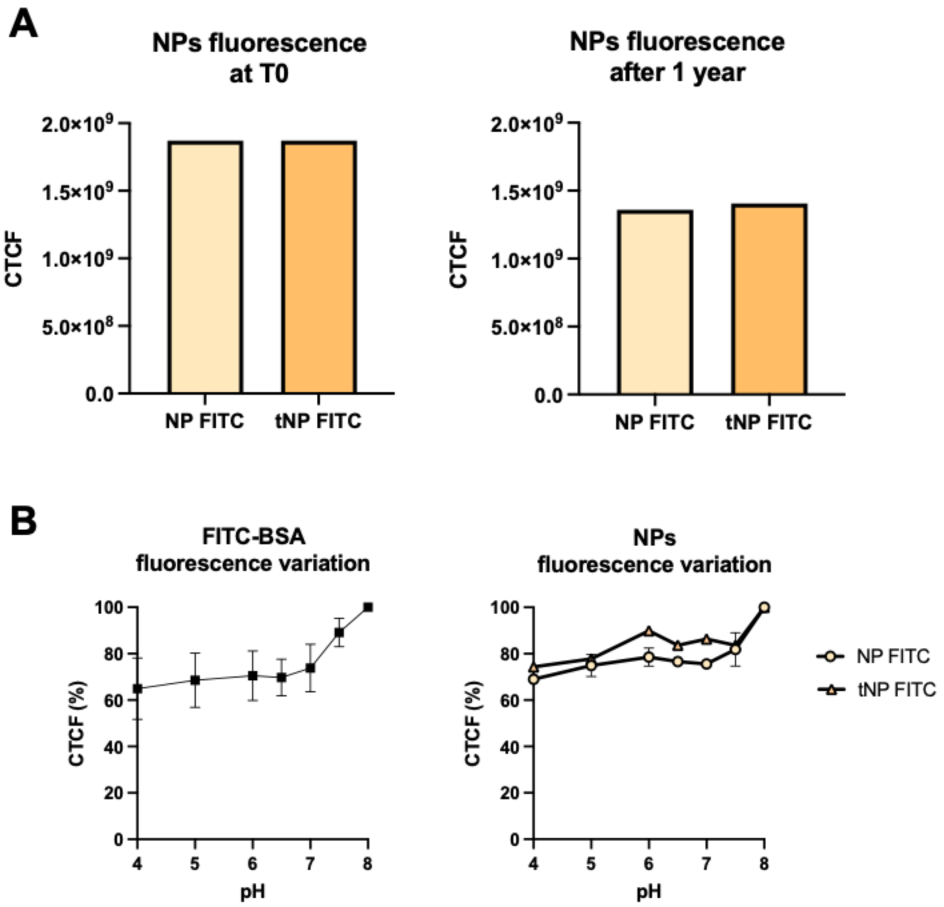

Supplementary Figure 1. Evaluation of NPs fluorescent properties. (A) Quantification of the fluorescent signal of FITC-BSA into NPs after synthesis (left) and after 1 year (right). Data are expressed as CTCF. (B) Fluorescence variation of FITC-BSA (left) and FITC-BSA loaded NPs (right) at different pH. Data are expressed as CTCF. The values are shown as mean ± SD of n=3.
